# Supplementary material for: Assessing the Reliability of SARS-CoV-2 Neutralization Studies That Use Post-Vaccination Sera
Source: Vaccines (Basel). 2022 May 26;10(6):850. doi: 10.3390/vaccines10060850 (PMC9227377; doi:10.3390/vaccines10060850)
Supplement: Supplementary file 1 [file vaccines-10-00850-s001.zip › vaccines-1702235-supplementary.pdf]

Assessing the Reliability of SARS-CoV-2 Neutralization Studies Using Post-Vaccination Sera

SUPPLEMENTARY DATA

Henning Jacobsen[1*†], Ioannis Sitaras[2*†], Marley Jurgensmeyer[3], Mick N. Mulders[4], David Goldblatt[5], Daniel R. Feikin[4], Naor Bar-Zeev[3], Melissa Higdon[3], Maria Deloria Knoll[3]

[1]Department of Viral Immunology, Helmholtz Centre for Infection Research, Braunschweig, Germany.

[2]W. Harry Feinstone Department of Molecular Microbiology and Immunology, Johns Hopkins Bloomberg School of Public Health, Baltimore, MD, USA.

[3]International Vaccine Access Center, Department of International Health, Johns Hopkins Bloomberg School of Public Health, Baltimore, MD, USA.

[4]Department of Immunizations, Vaccines and Biologicals, World Health Organization, Geneva, Switzerland.

[5]Great Ormond Street Institute of Child Health NIHR Biomedical Research Centre, University College London, London, UK.

*To whom correspondence should be addressed.

†These authors contributed equally.

Supplementary Table S1

COHORT DETAILS		
**SAMPLE SIZE**		
**Sample size**<br>*How many samples were included?*	Possible outcomes	Risk of bias
Required to assess the statistical strength, potential for spurious results and overall generalizability of results. Because that probability of spurious results can vary with technical quality of the study and degree of heterogeneity of the population from which specimens were selected with respect to confounding factors, a single threshold cannot be identified, but generally, larger N can mitigate these influences.<br>The reviewer should consider population heterogeneity and technical study quality to adjust the threshold and/or risk of bias for each study.	>50	NO
	21-50	LOW
	5-20	MEDIUM
	<5	HIGH
	Not reported	UNCLEAR
**SARS-COV-2 INFECTION**		
**Reported**<br>*Was pre-vaccination COVID-19 considered?*	Possible outcomes	Risk of bias
There is accumulating evidence that convalescent subjects develop a stronger immune response to vaccination compared to SARS-CoV-2 naïve subjects [1-3]. Therefore, it is important to provide sufficient information about pre-vaccination COVID-19 in the study population to consider this aspect as a potential risk of bias.<br>If no information on this aspect are available, the respective risk of bias is unclear. In early pandemic settings the likelihood of previous SARS-CoV-2 infection is small, therefore the risk of bias should be considered medium. We recommend using September 30th 2020 as a cut-off for early pandemic settings as Q4 in 2020 went in line with a drastic increase of cases worldwide and identification of first clinically-relevant variants [4, 5].<br>We recommend applying a high risk of bias for this criterion if it is reported and for ≥20% of the study cohort stratification or proper study design is missing.	Yes, only naïve included	NO
	Yes, and subjects stratified	NO
	Yes, but not stratified	HIGH
	Not reported and early pandemic setting	MEDIUM
	Not reported and late pandemic setting	UNCLEAR
**Confirmed**<br>*Previous infection confirmed by NP-ELISA or similar means?*	Possible outcomes	Risk of bias
Self-report is likely to have low sensitivity since not everyone would be tested, and asymptomatic infections may not trigger testing. Additional, self-reported negative test results may not reflect status at time of specimen collection. Because the potential impact of non-naïve subjects is high, the study population should be screened by the investigators for previous COVID-19 by highly sensitive methods (e.g., NP-ELISA or by repeated qPCR screening or antigen testing over the whole study period (and pre-study period if applicable). If the pre-vaccination COVID-19 status is reported but not confirmed, we assign a low risk of bias, because there is still a risk of unreported / unconfirmed cases although it is likely, that most cases are considered by the respective screening-method.	Yes	NO
	No / not reported	LOW
	N. a.	NO

Breakthrough cases reported<br>*Are breakthrough cases reported?*	Possible outcomes	Risk of bias
Especially in longitudinal studies, breakthrough cases of COVID-19 might appear in the study cohort. Similar to pre-vaccination infections, these infections are known to affect the subject's immune response and neutralization titers because of boosting-like effects. If breakthrough cases are likely to appear in the study setting (e.g. longitudinal studies), this can pose a significant bias affecting some or even several subjects. We therefore assign a medium risk of bias for this aspect, because of a strong effect that is yet likely to only affect a small subset of the study population.	Yes	NO
	No	MEDIUM
	N. a.	NO
**Breakthrough cases stratified**<br>*If breakthrough cases appeared, do the authors stratify?*	Possible outcomes	Risk of bias
If breakthrough cases of COVID-19 are reported for the study cohort, neutralization results should be stratified for naïve and infected subjects to acknowledge booster-effects of the infection. We recommend applying this criterion if for ≥20% of the study cohort breakthrough cases are reported. Missing stratification can result in a high risk of bias, dependent on the number of affected subjects.	Yes	NO
	No	HIGH
	N. a.	NO
**VACCINATION REGIMEN**		
**Dosing interval reported**<br>*Do the authors report the dosing interval (if applicable)?*	Possible outcomes	Risk of bias
There is increasing evidence that the dosing interval for vaccines with a prime-boost regimen can affect the immune response including neutralization titers [6, 7]. We therefore recommend considering the dosing interval in interpretation of the data and missing information as a medium risk of bias.	Yes	NO
	No	MEDIUM
	N. a.	NO
**Stratified by partial / full immunization**<br>*Do the authors stratify for partial and full immunization?*	Possible outcomes	Risk of bias
Certain studies investigate neutralization titers from partially and fully vaccinated individuals. It is imperative that these cohorts are completely separated, as it is known that post-prime titers are significantly inferior to post-boost titers. We assign a medium risk of bias for this aspect because most studies only include small numbers of partially immunized subjects into the respective study. Studies involving greater proportions of non-stratified subjects should not be considered.	Yes	NO
	No	MEDIUM
	N. a.	NO
**SAMPLE COLLECTION PERIOD**		
**≥ 7 days post last dose**<br>*Were all samples taken at least seven days post final dose?*	Possible outcomes	Risk of bias
Because of the kinetics of neutralizing antibody generation, no samples taken ≤ 7 days post immunization should be considered [8-10]. We recommend excluding any study involving ≥20 % of subjects with too early sampling from further consideration.	Yes	NO
	No	HIGH
	Not reported	UNCLEAR

**Stratified OR ≥ 14 days and ≤ 4 months post last dose**<br>*Are the results stratified OR are all samples taken ≥ 14 days and ≤ 4 months post final dose?*	Possible outcomes	Risk of bias
Peak neutralization titers are usually observed 14-28 days post immunization followed by a gradual decline of neutralization activity (waning) [8-10]. When assessing neutralization results and especially when comparing studies, it is important to acknowledge these kinetics by stratification of the results or by only including subjects sampled within a range of peak titers. Based on currently available literature, we defined 4 months post last dose as the upper limit for this period [8, 9]. Because (waning) kinetics of neutralizing antibodies against SARS-CoV-2 are not fully understood yet, we assign a medium risk of bias for this aspect. We recommend applying this criterion if for ≥20% of the study cohort stratification or proper study design is missing.	Yes	NO
	No	MEDIUM
	Not reported	MEDIUM
	N. a.	NO
**DEMOGRAPHIC CHARACTERIZATION**		
**Age distribution reported**<br>*Is the age distribution (range) of all subjects reported?*	Possible outcomes	Risk of bias
As for many other pathogens, age is very likely to also affect neutralization titers against SARS-CoV-2, especially when imperfect responses are reported [11-13]. Therefore, the age-structure of the study cohort should be reported to allow proper interpretation of results. Because the effect of age on anti-SARS-CoV-2 neutralization is not yet fully understood but age was shown to play an important role for other pathogens, we assign a medium risk of bias for this aspect.	Yes	NO
	No	MEDIUM
**Stratified by age group**<br>*Are results stratified by the subject's age?*	Possible outcomes	Risk of bias
To acknowledge possible effects of age on neutralization titers, we recommend stratifying results based on age groups, especially for older adults (≥ 60 years), adults and children (< 18 years), if ≥20 % of the study cohort belong to different age groups. We assign a low risk of bias for missing age-stratification if the age distribution is reported, because context-specific interpretation remains possible.	Yes	NO
	No	LOW
	Not reported	LOW
**Sex distribution reported**<br>*Is the sex distribution of all subjects reported?*	Possible outcomes	Risk of bias
Although there is conflicting data, several studies suggest that the biological sex might also affect neutralization titers against SARS-CoV-2. Therefore, consideration of sex might support correct interpretation of results [14, 15]. Because the effect of the biological sex on anti-SARS-CoV-2 neutralization is currently poorly understood, we assign a low risk of bias for this aspect.	Yes	NO
	No	LOW

**Stratified by sex OR equal sex distribution**<br>*Are results stratified by sex or are sexes represented equally?*	Possible outcomes	Risk of bias
To acknowledge possible effects of the biological sex on neutralization titers, we recommend stratifying results based on the subjects' sex. This aspect should not apply if the cohorts comprises an equal sex distribution (50% ±10% per sex) or if ≥90% of the cohort belong to one sex (assumption of almost sex-specific results). Because the effect of the biological sex on anti-SARS-CoV-2 neutralization is currently poorly understood, we assign a low risk of bias for this aspect.	Stratified	NO
	Equal distribution	NO
	Not stratified nor equally distributed	LOW

**Cohort selection unbiased**<br>*Do the authors select the subjects without bias?*	Possible outcomes	Risk of bias
In most studies, total IgG-titers are assessed along with neutralization titers. Some studies chose to then separate the study cohort into different categories of "responders" based on the antibody response for further analysis. If neutralization titers are generally assessed, it is essential, that no biased pre-selection was performed on the study cohort. Results restricted to example elite-responders or non-responders should not be considered for general use and analysis.	Yes	NO
	No	HIGH
	Not reported	UNCLEAR
**Study period and geographic location reported**<br>*Do the authors report study period and geographic location?*	Possible outcomes	Risk of bias
If pre-vaccination or post-vaccination (breakthrough) SARS-CoV-2 infections occurred during the study, it appears important to consider these events for the assessment of the neutralization response. For this, it is important to understand which SARS-CoV-2 variants caused infection, because there is increasing evidence, that variants can have differential effects on the neutralization response [16]. If the variant distribution is not provided within the manuscript, the study period and geographic location allows predicting a likely distribution of variants.<br>Because the effect of variant-specific infection on anti-SARS-CoV-2 neutralization is currently poorly understood, we assign a low risk of bias for this aspect.	Yes	NO
	No	LOW
	N. a.	NO
**Variant prevalence reported**<br>*Do the authors report the prevalence of variants (if applicable)?*	Possible outcomes	Risk of bias
As described above, the prevalence of variants can help to understand and to correctly interpret data in the context of SARS-CoV-2 infections that occurred during or before the study period. Only applicable if SARS-CoV-2 infections occurred. Because the effect of variant-specific infection on anti-SARS-CoV-2 neutralization is currently poorly understood, we assign a low risk of bias for this aspect.	Yes	NO
	No	LOW
	N. a.	NO
**Stratified by variant prevalence**<br>*Are results stratified by variant prevalence (if applicable)?*	Possible outcomes	Risk of bias
	Yes	NO

	Possible outcomes	Risk of bias
If a considerable amount (≥20%) of SARS-CoV-2 infections occurred during or before the study period, we recommend stratifying the results by the respective variants causing infection to acknowledge emerging data on potential effects of SARS-CoV-2 infection on cross-neutralization response in vaccinees [16]. Because the effect of variant-specific infection on anti-SARS-CoV-2 neutralization is currently poorly understood, we assign a low risk of bias for this aspect.	No	LOW
	N. a.	NO
**CLINICAL CHARACTERIZATION**		
**Reported**<br>*Do the authors report any relevant clinical characterization?*	Possible outcomes	Risk of bias
Many studies assess neutralization titers in groups of individuals that are likely to have clinical characteristics that might affect the post-vaccination immune response. Some examples are immuno-suppression (more likely in older adults), frailty (more likely in women) or pregnancy (women of reproductive age only). If it appears that the study cohort might consist of a relevant proportion (≥20%) of subjects that are likely to show immune-alternating clinical characteristics, the relevant clinical characteristics of the study cohort must be reported. If this information is missing, it is not possible to assess a related risk of bias and we recommend exclusion of this study from further analysis.	Yes	NO
	No	UNCLEAR
	N. a.	NO
**Stratified by immuno-compromised**<br>*Are the results stratified for immuno-compromised subjects?*	Possible outcomes	Risk of bias
If a clinical characterization is reported, we highly recommend stratifying the results for immuno-compromised subjects as they might significantly affect the overall neutralization titers in a cohort [17, 18]. Because of this, failure of stratification leads to a high risk of bias, yet we suggest that this aspect should only apply if ≥20% of the study cohort are eligible for stratification.	Yes	NO
	No	HIGH
	Not reported	UNCLEAR
	N. a.	NO
**ASSAY DETAILS**		
**PROTOCOL**		
**Assay type reported**<br>*Do the authors report the precise assay and endpoint?*	Possible outcomes	Risk of bias
To correctly interpret and compare studies, it is imperative that the assay type (live virus neutralization, pseudo virus neutralization; TCID, plaque-reduction neutralization etc.) along with the determined endpoint (NT20, NT50, NT80 etc.) is reported. There is by now increasing evidence that both the assay type as well as the endpoint can affect the neutralization titer [19-21]. If this information is missing, it is not possible to assess a related risk of bias and we recommend exclusion of this study from further analysis.	Yes	NO
	No	UNCLEAR
**Precise protocol reported**<br>*Do the authors provide a precise assay protocol?*	Possible outcomes	Risk of bias
A precise assay protocol can help to correctly interpret results and to understand possible differences among studies. Missing or low-quality	Yes	NO

information represent a risk of bias. We assign a low risk of bias to this aspect, because most studies provide at least essential information allowing for some extent of quality assessment.	No	LOW
**LIVE VIRUS STRAIN (IF APPLICABLE)**		
**Virus lineage reported**<br>*Do the authors provide the virus lineage and origin?*	Possible outcomes	Risk of bias
If a live virus is used for neutralization, the lineage and origin must be reported to allow correct interpretation of results. If this information is missing, it is not possible to assess a related risk of bias and we recommend exclusion of this study from further analysis.	Yes	NO
	No	UNCLEAR
	N. a.	NO
**Sequence confirmation by sequencing**<br>*Do the authors confirm the virus sequence by sequencing?*	Possible outcomes	Risk of bias
By now, there is substantial evidence that SARS-CoV-2 can acquire adaptational mutations in cell culture by serial passaging. Because it is currently not known if these mutations might affect neutralization titers, the virus sequence should be confirmed for the passage that is used for neutralization assays.<br>Because the potential impact of cell culture-adapting mutations on antibody neutralization is not fully understood, we assign a medium risk of bias for this aspect.	Yes	NO
	No	MEDIUM
	N. a.	NO

**PSEUDO VIRUS STRAIN (IF APPLICABLE)**		
**Construct details reported**<br>*Do the authors provide full construct details and origin?*	Possible outcomes	Risk of bias
If a pseudo virus is used for neutralization, details on pseudo virus construction and origin must be reported to allow correct interpretation of results. If this information is missing, it is not possible to assess a related risk of bias and we recommend exclusion of this study from further analysis.	Yes	NO
	No	UNCLEAR
	N. a.	NO
**All variant-associated spike mutations**<br>*Does the construct contain all variant-defining spike mutations?*	Possible outcomes	Risk of bias
To properly assess antibody neutralization against SARS-CoV-2 variants using a pseudo-virus system, it is important that the virus construct contains at least all spike-mutations that are associated to the respective variant. We recommend https://covdb.stanford.edu/ as a reference. If mutations are missing in the construct, this adds a medium risk of bias as the individual but also synergistic / antagonistic role of single mutations within a virus is currently poorly understood.	Yes	NO
	No	MEDIUM
	N. a.	NO
**Sequence confirmation by sequencing**<br>*Do the authors confirm the construct sequence by sequencing?*	Possible outcomes	Risk of bias

To follow good scientific practice and to provide maximum credibility of the assay, we recommend confirming the pseudo virus sequence (not the plasmids) by sequencing prior to use in neutralization assays. We assign a low risk of bias for missing sequencing information, because pseudo-viruses are artificially generated and commonly pre-constructs such as spike-expressing plasmids are sequence confirmed.	Yes	NO
	No	LOW
	N. a.	NO
**ASSAY STANDARDIZATION**		
**Virus titer reported and consistent**<br>*Are virus titers used for neutralization assays reported and if so: consistent and with small input variance?*	Possible outcomes	Risk of bias
With a neutralization assay, the capability of the subject's sample (usually plasma or serum) to neutralize a defined amount of virus is measured. Standardization of input-virus is essential to provide high-quality results. The variance that is accepted for the virus input directly translates into variance of the neutralization titer. Furthermore, the quantified virus input defines sensitivity and resolution of the assay. Therefore, we consider reporting the virus titer and use of a consistent amount of virus as essential and failure to do so as a high risk of bias. If this information is missing, it is not possible to assess a related risk of bias and we recommend exclusion of this study from further analysis.	Consistent and witch small variance	NO
	Not consistent or with high variance	HIGH
	Not reported	UNCLEAR
**Error in titer reported by back titration**<br>*Is the virus input confirmed by back-titration or similar means?*	Possible outcomes	Risk of bias
The virus input for each assay performed can be easily assessed by back titration. This allows to precisely describe the variance conferred by the virus input and therefore optimal assessment of the assay results. Because this is mainly an aspect for quality control, we assign a low risk of bias if this confirmation is missing.	Yes	NO
	No	LOW

**WHO international standard antibody used**<br>*Was the WHO IS standard antibody used for standardization?*	Possible outcomes	Risk of bias
By now, the WHO international standard antibody is available to allow standardization of neutralization results for SARS-CoV-2 neutralizing antibodies [22]. This standardization can enhance comparability of results and can support optimal interpretation of results, yet it is not essential, and lack of this information only possesses a low risk of bias.	Yes	NO
	No	LOW
**Details on cell culture reported**<br>*Do the authors provide precise details on cell culture?*	Possible outcomes	Risk of bias
Neutralization assays are performed in a cell culture environment and the final readout is remaining infectivity of non-neutralized virus. The infectivity is highly dependent on the target cells and can be influenced by many factors such as confluency, passage number, contamination, temperature and many more. We therefore recommend reporting cell culture techniques as detailed as possible to allow optimal interpretation of	Yes	NO
	No	LOW

results. Because this is mainly an aspect for quality control, we assign a low risk of bias if this confirmation is missing.		

**DATA**		
**DATA REPORTING**		
**Raw data reported**<br>Do the authors report raw data for neutralization titers?	Possible outcomes	Risk of bias
Direct reporting of raw data (ideally linked to the respective subject information such as age, sex etc.) supports optimal interpretation of results. Furthermore, raw data can be used to confirm or re-analyze statistics, if applicable. Because this is mainly an aspect for quality control, we assign a low risk of bias if this confirmation is missing.	Yes	NO
	No	LOW
**Reference virus is appropriate**<br>*Is the reference virus reasonable (if applicable)*	Possible outcomes	Risk of bias
In some studies, fold changes are calculated using the alpha variant as a reference. However, when using post-vaccination sera, it is important that comparisons are always made using the vaccine seed strain or a sufficiently similar strain as a reference, since the homologous comparison will determine the baseline neutralization activity of the sera, and any antigenic differences between the vaccine strain and other variants [23].	Yes	NO
	No	MEDIUM
	N. a.	NO
**Data shown as individual data points with statistics**<br>*Do the authors provide individuals data points and statistics?*	Possible outcomes	Risk of bias
Appropriate presentation of data and statistics can support correct interpretation of results and re-analysis as applicable. The sole presentation of for example fold-changes or bar graphs without presentation of data distribution adds uncertainty to the results and does not allow for optimal assessment. Because this is mainly an aspect for quality control, we assign a low risk of bias if this confirmation is missing.	Yes	NO
	No	LOW

Supplementary Data 2

See excel document

Supplementary Data 3

See excel document

Supplementary Data 4

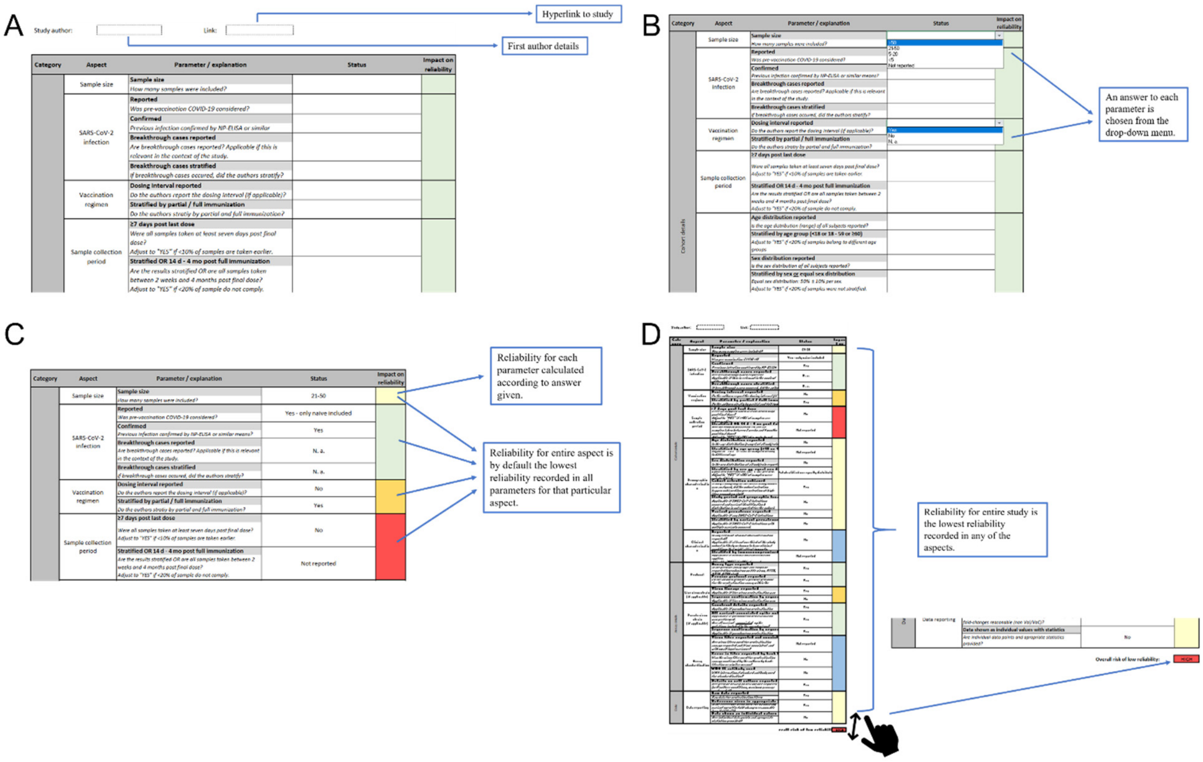

References

[1] Saadat S, Rikhtegaran Tehrani Z, Logue J, Newman M, Frieman MB, Harris AD, et al. Binding and Neutralization Antibody Titers After a Single Vaccine Dose in Health Care Workers Previously Infected With SARS-CoV-2. JAMA. 2021;325:1467-9.
[2] Stamatatos L, Czartoski J, Wan YH, Homad LJ, Rubin V, Glantz H, et al. mRNA vaccination boosts cross-variant neutralizing antibodies elicited by SARS-CoV-2 infection. Science. 2021.
[3] Vicenti I, Gatti F, Scaggiante R, Boccuto A, Zago D, Basso M, et al. Single-dose BNT162b2 mRNA COVID-19 vaccine significantly boosts neutralizing antibody response in health care workers recovering from asymptomatic or mild natural SARS-CoV-2 infection. Int J Infect Dis. 2021;108:176-8.
[4] Davies NG, Abbott S, Barnard RC, Jarvis CI, Kucharski AJ, Munday JD, et al. Estimated transmissibility and impact of SARS-CoV-2 lineage B.1.1.7 in England. Science. 2021;372.
[5] Galloway SE, Paul P, MacCannell DR, Johansson MA, Brooks JT, MacNeil A, et al. Emergence of SARS-CoV-2 B.1.1.7 Lineage - United States, December 29, 2020-January 12, 2021. MMWR Morb Mortal Wkly Rep. 2021;70:95-9.
[6] Payne RP, Longet S, Austin JA, Skelly DT, Dejnirattisai W, Adele S, et al. Immunogenicity of standard and extended dosing intervals of BNT162b2 mRNA vaccine. Cell. 2021;184:5699-714 e11.
[7] Flaxman A, Marchevsky NG, Jenkin D, Aboagye J, Aley PK, Angus B, et al. Reactogenicity and immunogenicity after a late second dose or a third dose of ChAdOx1 nCoV-19 in the UK: a substudy of two randomised controlled trials (COV001 and COV002). Lancet. 2021;398:981-90.
[8] Doria-Rose N, Suthar MS, Makowski M, O'Connell S, McDermott AB, Flach B, et al. Antibody Persistence through 6 Months after the Second Dose of mRNA-1273 Vaccine for Covid-19. N Engl J Med. 2021;384:2259-61.
[9] Collier AY, Yu J, McMahan K, Liu J, Chandrashekar A, Maron JS, et al. Differential Kinetics of Immune Responses Elicited by Covid-19 Vaccines. N Engl J Med. 2021;385:2010-2.
[10] Levin EG, Lustig Y, Cohen C, Fluss R, Indenbaum V, Amit S, et al. Waning Immune Humoral Response to BNT162b2 Covid-19 Vaccine over 6 Months. N Engl J Med. 2021.
[11] Bates TA, Leier HC, Lyski ZL, Goodman JR, Curlin ME, Messer WB, et al. Age-Dependent Neutralization of SARS-CoV-2 and P.1 Variant by Vaccine Immune Serum Samples. JAMA. 2021.
[12] Collier DA, Ferreira I, Kotagiri P, Datir RP, Lim EY, Touizer E, et al. Age-related immune response heterogeneity to SARS-CoV-2 vaccine BNT162b2. Nature. 2021;596:417-22.
[13] Muller L, Andree M, Moskorz W, Drexler I, Walotka L, Grothmann R, et al. Age-dependent immune response to the Biontech/Pfizer BNT162b2 COVID-19 vaccination. Clin Infect Dis. 2021.
[14] Grzelak L, Velay A, Madec Y, Gallais F, Staropoli I, Schmidt-Mutter C, et al. Sex Differences in the Evolution of Neutralizing Antibodies to Severe Acute Respiratory Syndrome Coronavirus 2. J Infect Dis. 2021;224:983-8.
[15] Markmann AJ, Giallourou N, Bhowmik DR, Hou YJ, Lerner A, Martinez DR, et al. Sex Disparities and Neutralizing-Antibody Durability to SARS-CoV-2 Infection in Convalescent Individuals. mSphere. 2021;6:e0027521.

[16] Liu C, Ginn HM, Dejnirattisai W, Supasa P, Wang B, Tuekprakhon A, et al. Reduced neutralization of SARS-CoV-2 B.1.617 by vaccine and convalescent serum. Cell. 2021;184:4220-36 e13.
[17] Haidar G, Agha M, Lukanski A, Linstrum K, Troyan R, Bilderback A, et al. Immunogenicity of COVID-19 Vaccination in Immunocompromised Patients: An Observational, Prospective Cohort Study Interim Analysis. medRxiv. 2021:2021.06.28.21259576.
[18] Galmiche S, Luong Nguyen LB, Tartour E, de Lamballerie X, Wittkop L, Loubet P, et al. Immunological and clinical efficacy of COVID-19 vaccines in immunocompromised populations: a systematic review. Clinical Microbiology and Infection.
[19] Riepler L, Rossler A, Falch A, Volland A, Borena W, von Laer D, et al. Comparison of Four SARS-CoV-2 Neutralization Assays. Vaccines (Basel). 2020;9.
[20] Sekirov I, Petric M, Carruthers E, Lawrence D, Pidduck T, Kustra J, et al. Performance comparison of micro-neutralization assays based on surrogate SARS-CoV-2 and WT SARS-CoV-2 in assessing virus-neutralizing capacity of anti-SARS-CoV-2 antibodies. Access Microbiology. 2021;3.
[21] von Rhein C, Scholz T, Henss L, Kronstein-Wiedemann R, Schwarz T, Rodionov RN, et al. Comparison of potency assays to assess SARS-CoV-2 neutralizing antibody capacity in COVID-19 convalescent plasma. J Virol Methods. 2021;288:114031.
[22] Kristiansen PA, Page M, Bernasconi V, Mattiuzzo G, Dull P, Makar K, et al. WHO International Standard for anti-SARS-CoV-2 immunoglobulin. Lancet. 2021;397:1347-8.
[23] Sitaras I. Antigenic Cartography: Overview and Current Developments. Methods Mol Biol. 2020;2123:61-8.
